# Expression and clinical significance of short-chain fatty acids in pregnancy complications

**DOI:** 10.3389/fcimb.2022.1071029

**Published:** 2023-01-12

**Authors:** Siqian Chen, Jialin Li, Shuaijun Ren, Yajie Gao, Yuping Zhou, Rongrong Xuan

**Affiliations:** ^1^ Department of Obstetrics and Gynecology, The Affiliated Hospital of Medical School, Ningbo University, Ningbo, China; ^2^ School of Medicine, Ningbo University, Ningbo, China; ^3^ Department of Gastroenterology, The Affiliated Hospital of Medical School, Ningbo University, Ningbo, China

**Keywords:** pregnancy complications, short-chain fatty acids, isobutyric acid, acetic acid, propionic acid, metabolites of intestinal flora

## Abstract

**Objective:**

To investigate the expression of short-chain fatty acids (SCFAs)—metabolites of intestinal flora—in gestational complications of gestational diabetes mellitus (GDM), preeclampsia (PE), and intrahepatic cholestasis of pregnancy (ICP), and its clinical significance.

**Methods:**

Targeted metabonomics was used to detect SCFAs in the serum of 28 GDM pregnant women, 28 PE pregnant women, 29 ICP pregnant women, and 27 healthy pregnant women (NP); their expression changes were observed; the correlation between SCFAs and clinical characteristics was studied; and their potential as biomarkers for clinical diagnosis was evaluated.

**Results:**

There were significant differences in the SCFA metabolic spectrum between the GDM, PE, ICP, and NP groups. Quantitative analysis showed that the content of isobutyric acid in the three pregnancy complications groups (the GDM, PE, and ICP groups) was significantly higher than that in the NP group (p < 0.05), and other SCFAs also showed significant differences in the three pregnancy complications groups compared with the NP group (p < 0.05). Receiver operating characteristic (ROC) curve analysis of the generalized linear model showed that multiple SCFAs were highly sensitive and specific as diagnostic markers in the pregnancy complications groups, where isobutyric acid was highly predictive in GDM (area under the ROC curve (AUC) = 0.764) and PE (AUC = 1), and caproic acid was highly predictive in ICP (AUC = 0.968), with potential clinical application.

**Conclusion:**

The metabolic products of intestinal flora, SCFAs, during pregnancy are closely related to pregnancy complications (GDM, PE, and ICP), and SCFAs can be used as potential markers of pregnancy complications.

## 1 Introduction

Pregnancy complications are caused by specific diseases associated with pregnancy or common diseases during pregnancy. The prevalence varies from 7.5% to 27.0% in different regions, mainly depending on ethnicities and diagnostic criteria (Soldavini et al., 2022). Gestational diabetes mellitus (GDM) increases the risk of gestational hypertension and preterm delivery, and it also causes disorders such as congenital malformations and hyperbilirubinemia in the offspring (Ye et al., 2022; Yan et al., 2022). Moreover, infants of GDM mothers are more likely to develop obesity and other metabolic disorders in adolescence and adulthood (Yuan et al., 2019). Preeclampsia (PE) is an important cause of maternal mortality with a prevalence of 2%–8% worldwide (Ozmen et al., 2022). Its pathogenesis has not been elucidated, but it is associated with systemic inflammation, endothelial function damage, and placental dysfunction (Melchiorre et al., 2022). Intrahepatic cholestasis of pregnancy (ICP) is a liver disease that develops during pregnancy and is characterized by increased maternal serum bile acid (BA) levels accompanied by pruritus and elevated liver enzymes (Dong et al., 2021). ICP is less harmful to the mother but increases the risk of adverse fetal outcomes, such as preterm delivery, amniotic fluid contamination, and fetal distress (Lin et al., 2022). So far, the pathophysiological mechanisms underlying these three disorders have not been fully elucidated. In recent years, the mechanism related to pregnancy complications and intestinal flora has been extensively studied. The intestinal flora and its metabolites can be used as new biomarkers, thus providing new ideas for the diagnosis and treatment of pregnancy complications (Chen et al., 2020; Zhan et al., 2021; Priyadarshini et al., 2022).

Short-chain fatty acids (SCFAs) are organic straight-chain carboxylic acids containing 1–6 atoms of carbon. They are the final products of indigestible carbohydrates formed by the fermentation by intestinal microbiota. The microbiota in the genital tract can also produce SCFAs (Amabebe and Anumba, 2020). Acetic acid, propionic acid, and butyric acid are the most abundant SCFAs (He et al., 2020). Approximately 500–600 mmol SCFAs are produced daily in the gut depending on diet, microbial composition, and residence time in the gut (Moniri and Farah, 2021). Dietary fiber is the main source of SCFAs in the intestinal tract. Most dietary fiber is not digested in the upper digestive tract, but it is fermented by different intestinal microorganisms after entering the cecum and colon. Among them, gram-negative bacteria (Bacteroides) mainly produce acetic acid and propionic acid, while gram-positive bacteria (Firmicutes) mainly produce butyric acid (He et al., 2020). In addition, Bacteroides and Clostridium can ferment branched-chain amino acids to produce branched-chain SCFAs (BSCFAs), including isobutyric acid and isovaleric acid (Zietek et al., 2021a). SCFAs can maintain the activity of intestinal mucosal immune cells and the integrity of intestinal epithelium, reduce the pH value of the colon, inhibit bacterial growth, and regulate energy metabolism, thereby playing an important role in hypertension, diabetes, cancer, and immune and metabolic diseases (Roy et al., 2020; Liu et al., 2021; Ratajczak et al., 2021). However, SCFAs produced by vaginal microbiota, especially acetic acid, can elicit diverse immunomodulatory effects on a range of cervicovaginal epithelial cell targets., which is a potential source of cervicovaginal inflammation in women (Aldunate et al., 2015; Delgado-Diza et al., 2020). Pregnancy can affect the composition of intestinal microbiota, and SCFAs produced by the microbial fermentation may also undergo different changes during pregnancy (Zietek et al., 2021b). SCFAs can affect the metabolic changes during pregnancy and the infant neurodevelopment (An et al., 2021; Hernandez-Martinez et al., 2022). It remains to be clarified how gut microbiota and SCFAs are involved in the development and progression of GDM, PE, and ICP, which are common diseases in pregnancy.

Therefore, based on the existing research progress on SCFAs and pregnancy-related diseases, in this study we used targeted metabolomics to carry out an analysis of serum SCFAs of pregnant women with GDM, PE, and ICP, and matched healthy control women, in order to explore the relationship between SCFAs and pregnancy-related diseases and related mechanisms, so as to provide further reference for the prevention, diagnosis, and intervention of pregnancy-related diseases.

## 2 Materials and methods

### 2.1 Participants

The subjects of this study were 112 pregnant women who gave birth in the Affiliated Hospital of Medical School of Ningbo University from October 2020 to December 2021, including 28 GDM patients, 28 PE patients, 29 ICP patients, and 27 healthy pregnant women (NP). The inclusion criteria were as follows: (1) singleton birth; (2) no history of hypertension, diabetes mellitus, cardio-cerebrovascular disease, and metabolic disease prior to pregnancy; (3) voluntarily participation and signed informed consent. The exclusion criteria were as follows: (1) the use of antibiotics, probiotics, and prebiotics within 1 month before sampling; and (2) the presence of diarrhea and other gastrointestinal symptoms. The diagnosis of GDM was based on the International Association of Diabetes and Pregnancy Study Group (IADPSG) criteria: oral glucose tolerance test (OGTT) performed between 24 and 28 weeks of gestation; fasting glucose ≥ 5.1 mmol/L (92 mg/dL); 1-hour-post-OGTT glucose ≥ 10.0 mmol/L (180 mg/dL) or 2-hour-post-OGTT glucose ≥ 8.5 mmol/L (153 mg/dL). The diagnosis of PE was based on the diagnostic standards of the American College of Obstetricians and Gynecologists (ACOG): (1) Gestational hypertension was defined as newly developed systolic and/or diastolic blood pressure ≥ 140/90 mm Hg after 20 weeks of pregnancy. The interval between two blood pressure measurements was at least 4 hours, and the postpartum blood pressure could return to normal. (2) Preeclampsia: 24-hour urine protein > 300 mg or protein/creatinine ≥ 0.3 mg/dL on the basis of hypertension in pregnancy; routine urine protein ≥ 2+; or negative urine protein consistent with the following new onset presentation: ①thrombocytopenia: platelet count < 100 × 109; ② renal insufficiency: serum creatinine > 1.1 mg/dL or twice higher than the upper limit of normal, excluding other kidney diseases; ③ impaired liver function: transaminases twice higher than the upper limit of normal; ④ pulmonary edema; and ⑤ new-onset headache not relieved by ordinary drug treatment, excluding other causes or blurred vision. ICP diagnosis was based on the diagnostic criteria for intrahepatic cholestasis of pregnancy formulated by the Chinese Medical Association: (1) skin itching that cannot be explained by other reasons; (2) fasting blood total BA levels ≥ 10 μmol/L; and (3) other causes of unexplained liver dysfunction, mainly mild-to-moderately elevated serum alanine aminotransferase (ALT) and aspartate aminotransferase (AST) levels, in the context of normal BA levels.

This study was approved by the Institutional Review Board (IRB) of the Affiliated Hospital of Medical School of Ningbo University (KY202011224). All of the participants signed a written informed consent form.

### 2.2 Collection of clinical data

We collected clinical information, including age, height, weight, pre-pregnancy BMI, blood pressure, gravidity, parity, gestational week of delivery, mode of delivery, and fetal birth weight of the four groups of subjects. Blood routine indicators, such as hemoglobin, white blood cell count, percentage (%) of neutrophils, and C-reactive protein level, as well as biochemical test results, such as blood glucose, triglycerides, total cholesterol, low-density lipoprotein, high-density lipoprotein, ALT, AST, and total BA levels, were collected.

### 2.3 Collection of biological samples

The blood samples of pregnant women were collected one week before birth. Cubital venous blood was collected from all of the participants after 8–10 hours of fasting. Blood was centrifuged (4°C, 4000 g, 7 min) within 2 hours at room temperature or 4 hours at 4°C, and subpackaged serum samples were obtained (each tube contained approximately 300 μL serum). The final serum samples were stored at −80°C until all of the samples were collected and uniformly sent for targeted metabolomics analysis.

### 2.4 Targeted metabolomic analysis of SCFAs

Quantification of seven SCFAs (acetate, propionate, butyrate, isobutyrate, valerate, isovalerate, and hexanoic acid) in the serum samples was performed. First, sufficient serum samples (20 μL) were mixed with 15% phosphoric acid (50 μL), 75 μg/mL internal standard solution (isocaproic acid, 10 μL), and ether (140 μL) for pretreatment, derivatization, and extraction of target analytes. The samples were centrifuged at 12000 g for 10 min at 4°C, and 150 μL of the upper organic layer was collected for analysis. The injection mode was split injection (10:1), and the carrier gas was helium (1 mL/min). SCFAs were analyzed using an column (30 m × 0.25 mm, 0.25 μm) with Electrospray ionization (ESI) source in the positive ionization mode. The small molecules were measured using gas chromatography-mass spectrometry (GC-MS). The temperatures of the chromatographic inlet, ion source, transfer line, and the quadrupole mass spectrometer were maintained at 250°C, 230°C, 250°C, and 150°C, respectively. The programmed temperature started at 90°C and increased to 120°C at a rate of 10°C/min, then to 150°C at a rate of 5°C/min. Finally, the temperature was increased to 250°C at a rate of 25°C/min for a period of 2 minutes.

### 2.5 Metabolomics data analysis

The potential differential metabolites were analyzed by bioinformatics methods including principal component analysis (PCA), partial least-squares discriminant analysis (PLS-DA), orthogonal partial-least squares discriminant analysis (OPLS-DA), and linear regression. Quantitative analysis of serum metabolites was performed with R through multivariate statistical analyses (version 3.1.3, pheatmap and ropls package, function cor, and cor. test). Pearson’s correlation coefficient or Spearman’s rank correlation coefficient was used to analyze the correlation among individual metabolites. In addition, receiver operating characteristic curve (ROC) analysis was performed, and the area under the curve (AUC) was used to evaluate the predictive diagnostic ability of the metabolites.

### 2.6 Statistical analysis

SPSS26.0 software (IBM, USA) was used for statistical analysis. Differences between groups were assessed with independent samples t-test for normally distributed data, and Wilcoxon rank-sum test for non-normally distributed data. Continuous variables were represented by mean ± standard deviation (SD). Chi-square test or the Fisher’s exact test was used for intergroup comparisons of categorical variables. A p-value < 0.05 was considered to be statistically significant.

## 3 Results

### 3.1 Clinical data of participants

A total of 112 women took part in this study, including 28 GDM, 28 PE, 27 ICP, and 29 NP women. The clinical characteristics of the mothers and children in the GDM group are shown in **Table 1**; those in the PE group are shown in **Table 2**; and those in the ICP group are shown in **Table 3**. The levels of fasting blood glucose and blood glucose at 1 and 2 hours after OGTT in the GDM group were significantly higher than those in the NP group (p < 0.05). Serum urea and urea/creatinine were also significantly higher than those in the NP group, while the white blood cell count was lower than that in the NP group (p < 0.05). There were no significant differences in blood lipids and liver function parameters (p > 0.05). The gestational weeks of delivery and fetal birth weight in the PE group were significantly lower than those in the NP group, while the cesarean section rate was significantly higher than that in NP group (p < 0.001). Systolic blood pressure and diastolic blood pressure were significantly higher (p < 0.001), and serum total BA, urea, and blood urea/creatinine were also significantly higher (p < 0.05) in the PE group than in the NP group, whereas white blood cell count, total cholesterol level, and albumin level were significantly lower (p < 0.05) in the PE group. Other maternal general characteristics and relevant laboratory data were not significantly different between the two groups (p > 0.05). The gestational weeks of delivery and fetal birth weight of the ICP group were also significantly lower than those of the NP group, and the cesarean section rate was significantly higher than that of the NP group (p < 0.001). In addition to total BA, maternal ALT and AST were also significantly higher in the ICP group than in the NP group, while white blood cell count, neutrophil percentage, hemoglobin, and albumin levels in the ICP group were lower (p < 0.05) than those in the NP group.

**Table 1:** Maternal and fetal clinical characteristics in the GDM and NP groups.

Variables	NP (n=27)	GDM (n=28)	*p*-value
Age (yrs)	28.30 ± 2.37	29.71 ± 4.35	0.104
Height (cm)	162.96 ± 5.21	161.00 ± 6.13	0.207
Prenatal BMI (kg/m^2^)	22.14 ± 2.66	21.58 ± 3.52	0.400
Weight gain (kg)	14.43 ± 5.00	14.42 ± 4.54	0.998
gravidity	2.00 ± 1.21	2.00 ± 1.36	0.717
Parity, n (%)			0.121
Primipara	16 (59)	22 (79)	
Multiparous	11 (41)	6 (21)	
Gestational age (weeks)	39.43 ± 0.82	39.40 ± 0.78	0.538
Delivery mode, n (%)			0.409
Vaginal delivery	21 (78)	19 (68)	
Cesarean delivery	6 (22)	9(32)	
Fetal birth weight (kg)	3.43 ± 0.43	3.45 ± 0.38	0.512
Blood pressure level			
Systolic blood pressure (mmHg)	120.67 ± 9.68	118.86 ± 10.34	0.506
Diastolic blood pressure (mmHg)	74.63 ± 6.66	73.61 ± 6.74	0.748
Blood routine index			
Hemoglobin (g/L)	127.96 ± 11.29	127.82 ± 11.04	0.963
White blood cells (*10^9/L)	11.10 ± 3.14	9.03 ± 2.26	**0.005**
Neutrophil percentage (%)	78.29 ± 6.48	75.49 ± 6.03	0.103
C reactive protein	5.83 ± 5.72	6.50 ± 10.21	0.296
Blood biochemical indicators			
Blood glucose level			
Fasting glucose, OGTT (mmol/L)	4.30 ± 0.33	4.65 ± 0.56	**0.008**
1 h glucose, OGTT (mmol/L)	8.04 ± 1.44	9.45 ± 1.61	**0.002**
2 h glucose, OGTT (mmol/L	6.98 ± 0.93	8.31 ± 1.55	**<0.001**
Triglycerides (mmol/L)	3.24 ± 0.98	3.23 ± 1.29	0.956
Total cholesterol (mmol/L)	6.49 ± 1.35	7.01 ± 1.03	0.111
Low density lipoprotein (mmol/L)	3.51 ± 0.87	3.82 ± 0.72	0.147
High-density lipoprotein (mmol/L)	2.06 ± 0.33	2.03 ± 0.39	0.711
Alanine aminotransferase (U/L)	9.44 ± 3.29	11.18 ± 4.52	0.148
Aspartate aminotransferase (U/L)	19.59 ± 3.63	20.00 ± 5.00	0.865
Albumin (g/L)	37.70 ± 2.25	36.76 ± 2.11	0.119
Total bile acids (μmol/L)	2.62 ± 1.37	3.31 ± 1.71	0.081
Urea (mmol/L)	3.24 ± 0.77	3.77 ± 0.95	**0.037**
Serum creatinine (μmol/L)	46.51 ± 6.22	47.11 ± 8.16	0.760
Urea/creatinine ratio	0.07 ± 0.02	0.08 ± 0.01	**0.015**
Serum uric acid (μmol/L)	333.07 ± 74.17	337.14 ± 75.33	0.827

The bold values are less than 0.05, which are statistically significant.

**Table 2:** Maternal and fetal clinical characteristics in the PE and NP groups.

Variables	NP (n=27)	PE (n=28)	*p*-value
Age (yrs)	28.30 ± 2.37	29.68 ± 4.67	0.174
Height (cm)	162.96 ± 5.21	161.89 ± 4.54	0.420
Prenatal BMI (kg/m^2^)	22.14 ± 2.66	22.84 ± 3.89	0.544
Weight gain (kg)	14.43 ± 5.00	14.66 ± 3.52	0.566
gravidity	2.00 ± 1.21	1.89 ± 1.29	0.523
Parity, n (%)			0.214
Primipara	16 (59)	21 (75)	
Multiparous	11 (41)	7 (25)	
Gestational age (weeks)	39.43 ± 0.82	38.00 ± 1.60	**<0.001**
Delivery mode, n (%)			**<0.001**
Vaginal delivery	21 (78)	8 (29)	
Cesarean delivery	6 (22)	20 (71)	
Fetal birth weight (kg)	3.43 ± 0.43	2.97 ± 0.62	**0.002**
Blood pressure level			
Systolic blood pressure (mmHg)	120.67 ± 9.68	145.86 ± 14.00	**<0.001**
Diastolic blood pressure (mmHg)	74.63 ± 6.66	93.71 ± 10.49	**<0.001**
Blood routine index			
Hemoglobin (g/L)	127.96 ± 11.29	126.29 ± 12.04	0.596
White blood cells (*10^9/L)	11.10 ± 3.14	8.80 ± 2.12	**0.002**
Neutrophil percentage (%)	78.29 ± 6.48	74.83 ± 6.84	0.06
C reactive protein	5.83 ± 5.72	4.092.60	0.494
Blood biochemical indicators			
Blood glucose level			
Fasting glucose, OGTT (mmol/L)	4.30 ± 0.33	4.37 ± 0.26	0.350
1 h glucose, OGTT (mmol/L)	8.04 ± 1.44	8.53 ± 1.06	0.297
2 h glucose, OGTT (mmol/L	6.98 ± 0.93	6.50 ± 0.87	0.051
Triglycerides (mmol/L)	3.24 ± 0.98	3.48 ± 1.01	0.429
Total cholesterol (mmol/L)	6.49 ± 1.35	5.40 ± 0.92	**0.001**
Low density lipoprotein (mmol/L)	3.51 ± 0.87	3.38 ± 0.65	0.529
High-density lipoprotein (mmol/L)	2.06 ± 0.33	2.09 ± 0.57	0.852
Alanine aminotransferase (U/L)	9.44 ± 3.29	11.54 ± 8.99	0509
Aspartate aminotransferase (U/L)	19.59 ± 3.63	20.57 ± 9.20	0.774
Albumin (g/L)	37.70 ± 2.25	35.49 ± 3.10	**0.004**
Total bile acids (μmol/L)	2.62 ± 1.37	4.32 ± 1.74	**<0.001**
Urea (mmol/L)	3.24 ± 0.77	4.00 ± 0.96	**0.002**
Serum creatinine (μmol/L)	46.51 ± 6.22	51.22 ± 11.00	0.189
Urea/creatinine ratio	0.07 ± 0.02	0.08 ± 0.02	**0.048**
Serum uric acid (μmol/L)	333.07 ± 74.17	361.18 ± 94.75	0.192

The bold values are less than 0.05, which are statistically significant.

**Table 3:** Maternal and fetal clinical characteristics in the ICP and NP groups.

Variables	NP (n=27)	ICP (n=29)	*p*-value
Age (yrs)	28.30 ± 2.37	29.83 ± 4.69	0.133
Height (cm)	162.96 ± 5.21	162.76 ± 4.75	0.879
Prenatal BMI (kg/m^2^)	22.14 ± 2.66	20.65 ± 3.65	**0.033**
Weight gain (kg)	14.43 ± 5.00	12.50 ± 5.69	0.186
gravidity	2.00 ± 1.21	2.07 ± 1.25	0.869
Parity, n (%)			0.103
Primipara	16 (59)	23 (79)	
Multiparous	11 (41)	6 (21)	
Gestational age (weeks)	39.43 ± 0.82	37.75 ± 1.73	**<0.001**
Delivery mode, n (%)			**<0.001**
Vaginal delivery	23 (77)	7 (24)	
Cesarean delivery	7 (23)	22 (76)	
Fetal birth weight (kg)	3.43 ± 0.43	3.06 ± 0.41	**0.002**
Blood pressure level			
Systolic blood pressure (mmHg)	120.67 ± 9.68	122.00 ± 6.25	0.540
Diastolic blood pressure (mmHg)	74.63 ± 6.66	74.97 ± 4.53	0.825
Blood routine index			
Hemoglobin (g/L)	127.96 ± 11.29	116.97 ± 12.71	**0.001**
White blood cells (*10^9/L)	11.10 ± 3.14	8.29 ± 2.43	**<0.001**
Neutrophil percentage (%)	78.29 ± 6.48	72.77 ± 9.41	**0.014**
C reactive protein	5.83 ± 5.72	3.30 ± 2.35	0.066
Blood biochemical indicators			
Blood glucose level			
Fasting glucose, OGTT (mmol/L)	4.30 ± 0.33	4.49 ± 0.55	0.125
1 h glucose, OGTT (mmol/L)	8.04 ± 1.44	7.53 ± 1.62	0.220
2 h glucose, OGTT (mmol/L	6.98 ± 0.93	6.51 ± 1.28	0.118
Triglycerides (mmol/L)	3.24 ± 0.98	3.19 ± 0.79	0.846
Total cholesterol (mmol/L)	6.49 ± 1.35	6.60 ± 1.32	0.481
Low density lipoprotein (mmol/L)	3.51 ± 0.87	3.28 ± 0.56	0.254
High-density lipoprotein (mmol/L)	2.06 ± 0.33	2.08 ± 0.46	0.884
Alanine aminotransferase (U/L)	9.44 ± 3.29	34.36 ± 46.10	**0.001**
Aspartate aminotransferase (U/L)	19.59 ± 3.63	34.71 ± 30.73	**0.014**
Albumin (g/L)	37.70 ± 2.25	35.47 ± 3.57	**0.008**
Total bile acids (μmol/L)	2.62 ± 1.37	25.88 ± 17.82	**<0.001**
Urea (mmol/L)	3.24 ± 0.77	3.40 ± 1.29	0.954
Serum creatinine (μmol/L)	46.51 ± 6.22	49.00 ± 12.41	0.896
Urea/creatinine ratio	0.07 ± 0.02	0.07 ± 0.02	0.700
Serum uric acid (μmol/L)	333.07 ± 74.17	330.01 ± 98.81	0.818

The bold values are less than 0.05, which are statistically significant.

### 3.2 Metabolomic profiling of serum SCFAs in pregnant women with pregnancy complications and normal pregnancies

**Figure 1:**
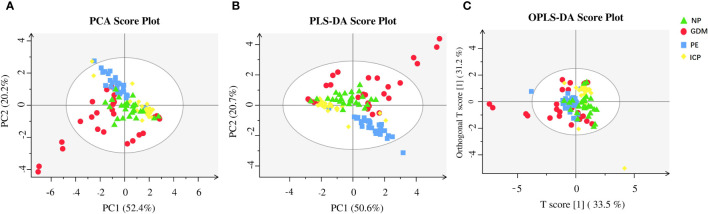
Metabolic analysis of SCFAs in pregnancy complications and NP groups. **(A)** PCA; **(B)** PLS-DA; **(C)** OPLS-DA. The abscissa is the first principal component (PC1), the ordinate is the second principal component (PC2), and the numbers in parentheses represent the proportion of the corresponding principal components in the comprehensive original information. One point in the figure corresponds to one sample, green represents the NP group, red represents the GDM group, blue represents the PE group, and yellow represents the ICP group.

**Figure 2:**
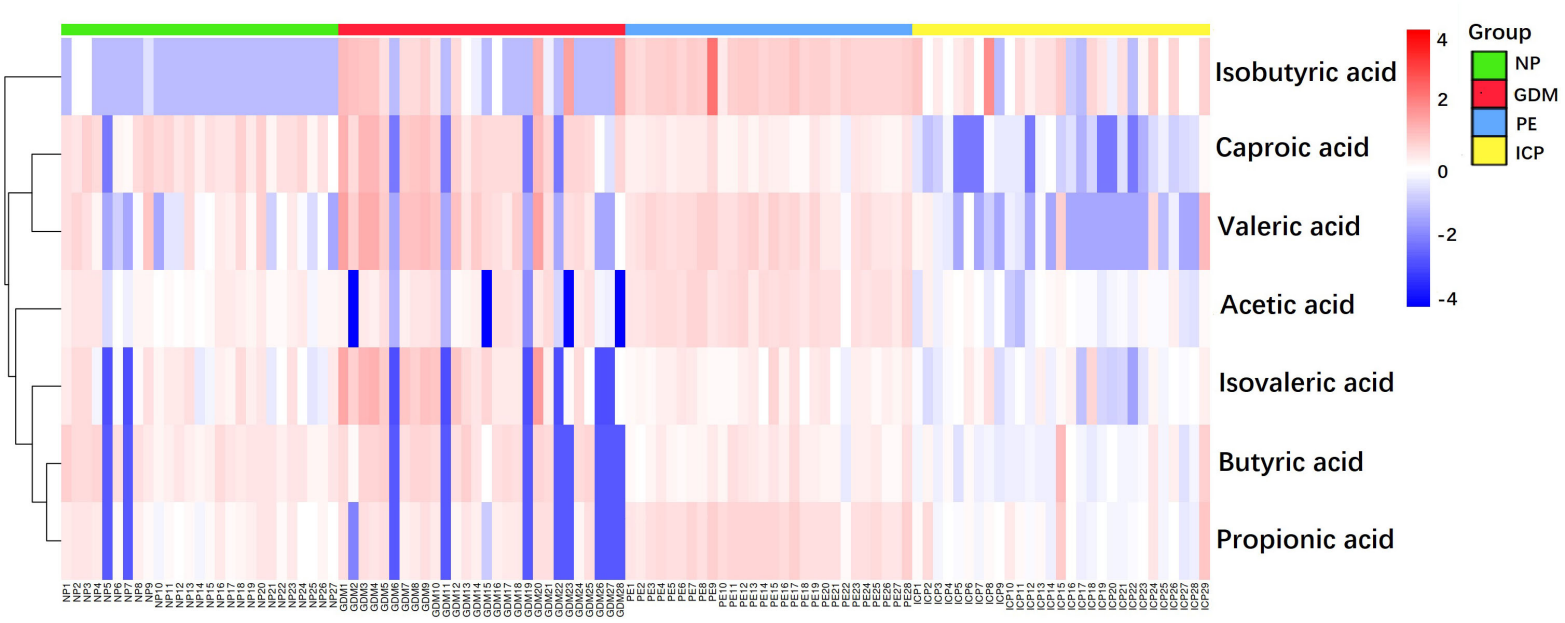
Heat map of SCFAs metabolites in pregnancy complications and NP groups. The magnitude of the relative amounts is shown by the difference in color, where the columns represent samples, and the rows represent SCFAs. Different colors represent different intensities, with red indicating relatively high kurtosis values and blue indicating relatively low kurtosis values.

In this study, PCA was used as an unsupervised pattern-recognition method to analyze the data of women with GDM, PE, and ICP, and their corresponding controls, and the results are shown in **Figure 1A**. We observed a clear separation between the pregnancy complications groups and the NP group. The supervised multivariate statistical analysis methods, PLS-DA and OPLS-DA, were used to further verify the statistical variability among the samples. A clear separation was visible in each sample and well-clustered within the same group, indicating differences in SCFAs among the groups (**Figures 1B, C**). To demonstrate the relationship of different samples more clearly and comprehensively, and thus evaluate the differences in metabolite expression patterns among different samples, agglomerative hierarchical clustering analysis was performed for each group of samples, so as to help us screen marker metabolites accurately and explore the changes of related metabolic processes. A heat map of metabolites was generated for the three pregnancy complication groups and the healthy pregnancy group (**Figure 2**). Isobutyric, isovaleric, valeric, and hexanoic acids were significantly higher in the GDM group than in the NP group. Acetic, propionic, isobutyric, and valeric acids levels were significantly higher in the PE group. In the ICP group, isobutyric acids levels were higher, while acetic, propionic, butyric, isovaleric, valeric, and hexanoic acids levels were significantly lower. These SCFAs showed the ability to distinguish between the two groups.

### 3.3 Change trends of serum SCFAs in pregnant women with pregnancy complications

**Figure 3:**
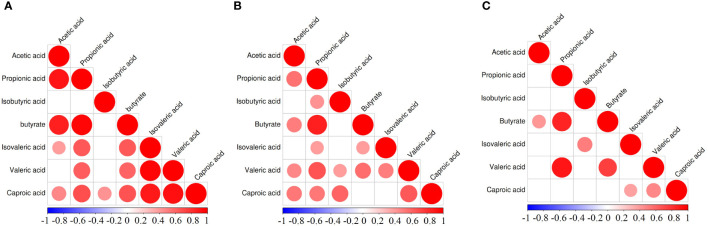
Correlation heatmap of different SCFAs in pregnancy complication groups. **(A)** GDM group, **(B)** PE group, **(C)** ICP group. When the linear relationship of the two metabolites is enhanced, the correlation coefficient tends to 1 or −1. The correlation is a maximum of 1, a complete positive correlation (red), a correlation of −1, and a complete negative correlation (blue). The white/clear color on the scale bar indicates no correlation.

The correlation heat map of seven SCFAs of each group of diseases showed correlations between various SCFAs (**Figure 3**). In the GDM group, hexanoic acid positively correlated with all of the SCFAs, and acetic acid and propionic acid positively correlated with butyric acid and isovaleric acid (**Figure 3A**). In the PE group, propionic acid and butyric acid showed a significant positive correlation, and valeric acids and other six SCFAs showed a positive correlation. Butyric acid and caproic acid positively correlated with acetic acid and propionic acid (**Figure 3B**). In the ICP group, propionic acid, butyric acid, and valeric acid showed significant positive correlations. Additionally, acetic acid and butyric acid; isovaleric acid and isobutyric acid; and caproic acid, isovaleric acid, and valeric acid also had positive correlations (**Figure 3C**).

### 3.4 Analysis of SCFAs expression in the pregnancy complications groups and the NP group

**Figure 4:**
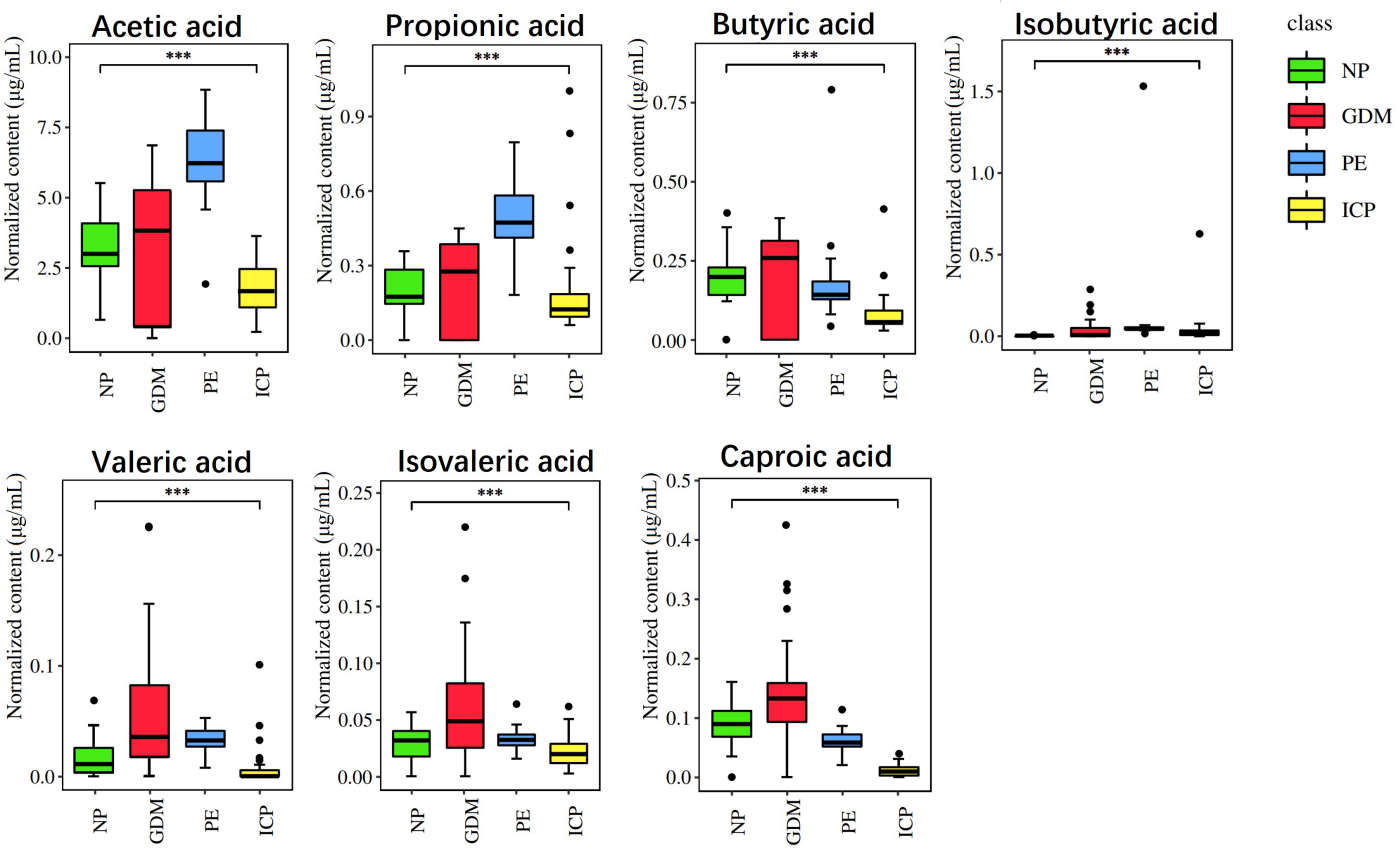
Quantitative analysis of SCFAs in pregnancy complications and NP groups. The abscissa is different groups, the ordinate is the content of short chain fatty acids, and different colors represent different groups. ***P < 0.001.

Quantitative analysis was carried out on seven SCFAs among the GDM, PE, ICP, and NP groups, and the differences between each two groups were analyzed. The results are shown in **Figure 4**. There were significant differences in SCFAs between the groups (p < 0.001), where isobutyric acid was significantly higher in the pregnancy complications groups (p < 0.001). In the GDM group, compared with the healthy pregnancy group, the contents of various SCFAs were higher (p < 0.001), with isobutyric acid being the most significantly different (67.39-fold of the NP group), followed by valeric acid (3.52-fold of the NP group). Levels of acetic, propionic, isobutyric, valeric, and isovaleric acid were higher in the PE group, where isobutyric acid level was 157.63 times higher and propionic acid level was 2.44 times higher compared with the NP group, while butyric acid and hexanoic acid levels were lower (p < 0.001). In the ICP group, the level of isobutyric acid was higher, 65.73 times that of the NP group, and the levels of acetic, propionic, butyric, isovaleric, valeric, and hexanoic acids were significantly lower compared with the NP group (p < 0.001).

### 3.5 Correlation between SCFAs and clinical parameters

**Figure 5:**
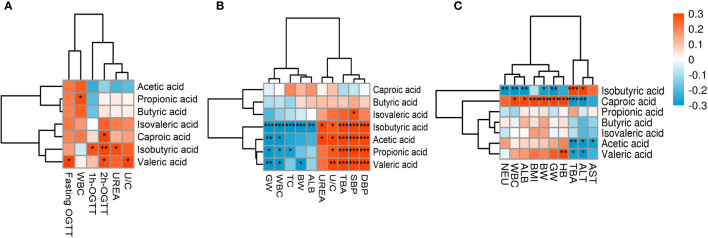
Heat map of correlations between SCFAs and clinical indicators. Red and blue squares represent positive and negative correlations, respectively. Statistical significance is indicated on the square (*P < 0.05; **P < 0.01; ***P < 0.001). WBC, White blood cells; UREA, Urea; U/C, Urea/creatinine ratio; GW, Gestational week; TC, Total cholesterol; BW, Birth weight; ALB, Albumin; TBA, Total bile acid; SBP, Systolic blood pressure; DBP,Diastolic blood pressure; NEU, Neutrophil percentage; BMI, Prenatal BMI; HB, Hemoglobin; ALT,Alanine aminotransferase; AST, Aspartate aminotransferase.

The statistically significant clinical indicators between the pregnancy complications groups and the healthy pregnancy group were correlated with SCFAs—the metabolites of gut flora—and the results are shown in **Figure 5**. In the GDM group, isobutyric acid significantly correlated with urea and blood glucose at 1 and 2 hours after OGTT; fasting blood glucose, blood glucose at 2 hours after OGTT, and urea/creatinine ratio positively correlated with valeric acid; blood glucose at 2 hours after OGTT positively correlated with caproic acid, and white blood cell count positively correlated with propionic acid (*p* < 0.05) (**Figure 5A**). In the PE group, the systolic blood pressure, diastolic blood pressure, urea/creatinine ratio, and total BA of pregnant women significantly positively correlated with acetic, propionic, isobutyric, and valeric acids; urea positively correlated with acetic and isobutyric acids; white blood cell count and gestational age negatively correlated with acetic, propionic, isobutyric, and valeric acids; total cholesterol negatively correlated with propionic and isobutyric acids (*p* < 0.05); and the percentage of neutrophils and fetal birth weight negatively correlated with isobutyric and valeric acids (*p* < 0.01) (**Figure 5B**). In the ICP group, isobutyric and hexanoic acids showed significant correlations with each clinical indicator (*p* < 0.05). Hemoglobin, white blood cells count, albumin, gestational weeks at delivery, fetal birth weight, and BMI were positively correlated with hexanoic acid, while total BAs and ALT were negatively correlated with hexanoic acid, and isobutyric acid showed the opposite trend with hexanoic acid. Valeric acid positively correlated with hemoglobin (*p* < 0.01), and it negatively correlated with total BA and ALT (*p* < 0.05) (**Figure 5C**). These results indicate that SCFAs are closely related to maternal clinical characteristics.

### 3.6 Analysis of the clinical diagnostic value of SCFAs

**Figure 6:**
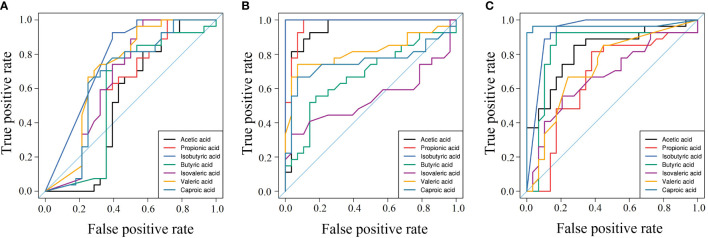
Area under the curve of pregnancy complications groups. **(A)** GDM group, **(B)** PE group, **(C)** ICP group. ROC curves for different metabolite combinations. AUC, Area under the curve. AUC>0.5, indicating that it has high predictive ability.

The AUC of SCFAs in pregnant women was used to evaluate their sensitivity and specificity in diagnosing the three diseases (GDM, PE, and ICP), and the results are shown in **Figure 6**. In the GDM group, the AUC was 0.514–0.764, and isobutyric and valeric acids had diagnostic potential for GDM; specifically, isobutyric acid had better diagnostic value (AUC = 0.764) and may be a serum biomarker for the diagnosis of GDM (**Figure 6A**). In the PE group, the AUC ranged from 0.543 to 1; and acetic, propionic, isobutyric, valeric, and hexanoic acids may be potential biomarkers for PE. Isobutyric acid showed the best diagnosability (AUC = 1), and propionic acid also had high diagnosability (AUC = 0.976) (**Figure 6B**). In the ICP group, the AUC ranged from 0.690 to 0.968; and acetic, butyric, isobutyric, valeric, and hexanoic acids showed the potential to diagnose ICP, with hexanoic acid showing high diagnostic accuracy (AUC = 0.968) (**Figure 6C**).

## 4 Discussion

GDM, PE, and ICP are common complications in pregnancy. They can lead to various adverse outcomes in pregnant women and newborns due to complex pathogenesis and diverse etiologies, and there is currently no effective treatment. It is of great significance to find related markers for early diagnosis and intervention. As important metabolites of intestinal microbiota, SCFAs have been extensively studied in recent years. SCFAs are mainly fermented by gut microbiota, which are differently altered as pregnancy progresses. Previous studies have shown that the gut microbiota is strongly associated with pregnancy complications (Chang et al., 2020; Chen et al., 2021; Zietek et al., 2021a). However, few studies have been conducted on SCFAs—the metabolites of intestinal flora. In this study, targeted metabonomic analysis was used to confirm that SCFAs are related to the progression of GDM, PE, and ICP. Quantitative analysis showed that compared with NP women, the content of isobutyric acid in the pregnancy complications groups was significantly higher, and the levels of other SCFAs were also significantly different from those in the NP group. ROC curve analysis showed that the sensitivity and specificity of acetic, propionic, and isobutyric acids in the PE group were high (AUC > 0.9), which is consistent with a previous study (Li et al., 2022). In addition, we also showed that SCFAs were closely related to the increased risk of ICP, and ROC curve analysis showed that caproic acid and isobutyric acid might be potential biomarkers (AUC > 0.9). Further analysis showed that SCFAs significantly correlated with the general characteristics of the mothers and various clinical indicators, so they are expected to be new biomarkers for clinical diagnosis and monitoring. To the best of our knowledge, this is the first retrospective study that links SCFAs to the risks of three types of pregnancy complications.

In this study, the content of isobutyric acid was significantly increased in all three pregnancy complications, and ROC curve analysis indicated that isobutyric acid may be a potential biomarker. The isovaleric acid level also significantly increased in GDM and PE patients. Both isobutyric and isovaleric acids are BSCFAs, which are produced by fermentation of branched-chain amino acids by intestinal flora. Milk and dairy products are unique dietary sources of BCFAs (Taormina et al., 2020). Higher levels of BCFAs are produced when eating a high-protein/low-carbohydrate diet (Zietek et al., 2021a). However, the influence of dietary factors needs further study. It has been found in mouse experiments that BCFAs increase glucose production in hepatocytes and activate the mTORC1/S6K1 signaling pathway to exacerbate diet-induced obesity and insulin resistance (Choi et al., 2021). Significantly elevated isobutyric acid and isovaleric acid in pregnant women with GDM may be associated with insulin resistance caused by BCFAs. However, another study suggested that BCFAs have strong anti-inflammatory potential and may have a beneficial effect on insulin sensitivity in humans (Su et al., 2021). BCFAs can reduce the expression level of genes encoding proinflammatory proteins in a dose-dependent manner to play an anti-inflammatory role (Czumaj et al., 2022). Yet, BSCFAs have been found to activate rapamycin target proteins (mTOR) in mammals and upregulate nuclear factor-κB (NF-κB) signaling pathways, increasing the release of proinflammatory cytokines in endothelial cells (Ye et al., 2020). The increase of BSCFAs in PE patients found in our study may also be involved in the release of proinflammatory cytokines.

The content of acetic and propionic acids in GDM and PE patients increased significantly. The ROC curve analysis showed that acetic and propionic acids had high diagnostic value for PE (AUC > 0.9). Acetic and propionic acids produced by gram-negative bacteria are abundant in the human body, and both are associated with lipid metabolism (Zietek et al., 2021a). The analysis of clinical data showed that cholesterol content in PE patients was significantly lower than that in the NP group. The correlation heat map further confirmed that cholesterol was negatively correlated with acetic acid and propionic acid levels. Another study found that increased adipose tissue and cholesterol in pregnant women promoted metabolic abnormalities and systemic inflammation, leading to poor placental vascularization and promoting the occurrence of PE (Alston et al., 2022). The reason for this difference may be related to dyslipidemia of pregnancy (DLP) caused by insulin resistance, increased lipoprotein synthesis, and lipolysis during normal pregnancy in the NP group (Gao et al., 2022). The content of acetic and propionic acids in GDM patients was also higher than that in the NP group, while cholesterol, triglycerides, and low-density lipoproteins showed no significant difference compared with the NP group, which might also be related to DLP. The correlation heat map between SCFAs and clinical indicators showed that propionic acid and leukocytes positively correlated in GDM, indicating that inflammation may mediate abnormal lipid metabolism through SCFAs and participate in the occurrence and development of GDM.

Butyric acid produced by gram-positive bacteria also plays an important role in regulating lipid disorders. Butyric acid can inhibit inflammation and lipid production by upregulating human antigen R (HuR) and inactivating the AMPK pathway (Su et al., 2021). This study showed that butyric acid content increased in the GDM group, which may be related to the lower triglyceride content in GDM patients. However, butyric acid content in the PE group decreased. Altemani et al. found that the number of butyrate-producing bacteria in the intestinal microbiota of pregnant women with preeclampsia decreased, and butyrate-production capacity and butyric acid level decreased (Altemani et al., 2021), which is consistent with the results of this study. Yong et al. fed sodium butyrate to preeclampsia rats, and they showed that it could improve their symptoms by reducing placental inflammation and maternal immune system disorder, and regulating the balance of placental angiogenic factors and anti-angiogenic factors (Yong et al., 2022). Hence, butyric acid has a beneficial effect on the regulation of blood pressure in preeclampsia.

In this study, only isobutyric acid was significantly increased, while other SCFAs were decreased in ICP patients, which may be related to the decreased abundance of flora in ICP patients. A previous study showed that BAs were blocked in the intestinal lumen with BA-chelating agents, and the contents of valeric acid and caproic acid were elevated (Li et al., 2021). Consistent with that study, the correlation heat map between SCFAs and clinical indicators in ICP patients in this study showed that acetic, valeric, and hexanoic acids negatively correlated with total BAs. This illustrated that SCFAs, including valeric and hexanoic acids, produced by gut flora were closely related to the occurrence and development of ICP. In particular, hexanoic acid (AUC > 0.9) was highly predictive of ICP. The heat map of correlations between SCFAs and clinical indicators showed a positive correlation between hexanoic acid content and leukocytes in ICP patients. Intestinal flora can promote cholestasis-mediated cell death and inflammation through macrophage activation of the inflammatory mechanism (Isaacs-Ten et al., 2020; Dreisbach et al., 2020). Caproic acid from intestinal flora may be related to inflammation in ICP patients. Another study showed that valeric acid and caproic acid can provide energy for the intestinal epithelium and play an anti-inflammatory role (Luu et al., 2019). In this study, the level of valeric acid in GDM and PE patients increased, and the content of valeric acid in PE patients negatively correlated with white blood cell count, further indicating that valeric acid may participate in inflammation-related metabolic abnormalities during pregnancy.

We found that the fetal birth weight of newborns whose mothers were PE and ICP patients was significantly lower than that of NP women, and PE may be related to the lower gestational age. There was no significant difference between ICP patients and the NP group in weight gain during pregnancy and gestational weeks. The BMI of ICP patients before pregnancy was lower than that of NP women, indicating that fetal birth weight may be related to excessive weight control of pregnant women. The relationship between pregnancy weight and ICP needs further study. Ma et al. found that participants who gained excessive weight during pregnancy had a higher abundance of Akkermansia muciniphila in early pregnancy (Ma et al., 2018). The protein Amuc_1100 isolated from Akkermania muciniphila can interact with Toll-like receptor 2 to repair the intestinal barrier and improve obesity and metabolic disorder (Plovier et al., 2017; Depommier et al., 2019). Thus, probiotics may reduce the enrichment of pathogenic bacteria in the intestinal tract and improve metabolic disorder during pregnancy. Propionic acid is the main metabolite of Akkermania muciniphila (Zhao and Li, 2017), which may be related to weight regulation during pregnancy. In addition, among various clinical indicators, urea and urea/creatinine in GDM and PE patients were significantly higher than those in the NP group, while white blood cell count was lower than that in NP women, and cholesterol content in PE patients was lower. These findings help to establish the risk of pregnancy complications, so as to actively take measures to improve perinatal outcomes.

This study has some limitations. First, SCFAs are produced from the food in the intestinal cavity during fermentation by intestinal microbiota. The effects of diet type, geographical environment, lifestyle, and drug factors on intestinal microbiota will likely affect the generated metabolites. The microbiota in the genital tract will also affect the production of metabolites. Although we adjusted for potential confounders in our analysis, other possible interfering factors cannot be excluded. Second, we chose pregnant women in the third trimester as the study subjects, and the intestinal flora may change at different stages of pregnancy, which limits the universality of our findings. Third, our study had a small sample size, which may have affected the stability of the outcome, so larger clinical samples are needed in the future to further investigate maternal SCFA levels during pregnancy and study the associated molecular mechanisms to determine their predictive value for pregnancy outcomes. Finally, this article only studies the metabolites in serum. In the follow-up study, we will further study the fecal metabolome to explore the correlation between microbiota and metabolome.

## Data availability statement

The raw data supporting the conclusions of this article will be made available by the authors, without undue reservation.

## Ethics statement

The studies involving human participants were reviewed and approved by the Institutional Review Board (IRB) of the Affiliated Hospital of Medical School of Ningbo University (KY202011224). The patients/participants provided their written informed consent to participate in this study.

## Author contributions

RX developed the concept. SC, JL, SR, and YG conducted the experiments and the statistical analysis. SC and JL organized the structure and wrote the manuscript. YZ and RX revised the manuscript. All authors contributed to the article and approved the submitted version.
